# Regional Effects of Maternal Mortality Determinants in Africa and the Middle East: How About Political Risks of Conflicts?

**DOI:** 10.3389/fpubh.2022.865903

**Published:** 2022-05-16

**Authors:** Jamal Mamkhezri, Somayeh Razzaghi, Mohsen Khezri, Almas Heshmati

**Affiliations:** ^1^Department of Economics, Applied Statistics and International Business, New Mexico State University, Las Cruces, NM, United States; ^2^Assistant Professor of Economics, Faculty of Economics and Social Sciences, Bu-Ali Sina University, Hamadan, Iran; ^3^Department of Economics and Finance, School of Management and Economics, University of Kurdistan Hewlêr (UKH), Erbil, Iraq; ^4^Professor of Economics, Jönköping International Business School, Jönköping University, Jönköping, Sweden

**Keywords:** maternal mortality, political risks, conflicts, Asia, Middle East

## Abstract

**Background:**

As per the United Nations Women data, the maternal mortality rate in war-affected countries is critical and more than 800 million people live in war-affected countries (ICRC). External and internal conflicts such as foreign pressure, war and cross-border, civil disorder, terrorism, and civil war, are characteristics of Middle Eastern and African countries. Therefore considering the rapid increment of political risks and internal and external conflicts in Africa and the Middle East during the last decade, and considering warfare as a key contributor to maternal mortality; This paper seeks to evaluate the factors that have caused significant rates of maternal mortality in Middle Eastern and African countries by emphasizing the contributions of a number of political risk aspects as Civil Disorder Index, Terrorism Index, Civil War Index, Foreign Pressures Index, Cross-Border Conflict Index, War Index along with other socio-economic factors.

**Method:**

Data were collected from forty-six countries during 2011–2016 to explore the regional contributions of political risk aspects to the maternal mortality rate through spatial approaches.

**Results:**

It was found that GDP per capita, energy intensity, and urbanization strongly impacted maternal mortality. Also, it was observed that natural resource rents and economic growth significantly influenced the reduction of mortality by expanding healthcare services. The urban expansion was found to have elevated maternal mortality. A majority of external and internal conflicts reduced the orientation of production toward healthcare services and thus raised maternal mortality. On the other hand, war and cross-border were found to pose opposite impacts.

**Conclusion:**

The findings revealed that political risks arising from terrorism, foreign pressure, and war in the adjacent countries would elevate the rate of mortality in the original country. This implies the spillover impacts of regional conflicts on maternal mortality elevation at the regional scale.

JEL Classification Codes: C23; I10; I18; N37:

## Introduction

Many factors could affect population health status rather than the national health system, such as socio-economic factors, gender inequality, natural disasters, and political unrest (e.g., warfare). Researchers opine that warfare has deeper and adverse effects on population health status than natural catastrophes (1). Diseases, malnutrition, and the destruction of health infrastructures are the unpleasant effects of warfare. In addition, studies show that females are more vulnerable than males to war, and women are at a higher risk of death due to the indirect effects of conflicts and poor health conditions in the Post-war period (2, 3). Bendavid et al. (4) state that Women of reproductive ages living near high-intensity conflicts have three times higher mortality than their counterparts in peaceful settings. Obermeyer et al. (5) demonstrate that women accounted for over 80 percent of reported war fatalities. Internal and external conflicts restrict maternal health and significantly harm the wellbeing of mothers by shrinking the availability of public and private health care services. According to the world health organization (WHO), 75% of countries observing a high maternal mortality rate in the world are war-affected, and in the year 2008, 50% of total women who died in childbirth were originally from only eight countries involving with ongoing or recent armed conflicts (6). In addition, O'Hare and Southall's (7) research in Africa showed that maternal mortality ratios in war-torn countries were 45% higher than in Non-conflict countries.

Although, warfare is a key contributor to maternal mortality; however, political risks of conflicts have increased rapidly in the last decade, especially in the Middle East and Africa. These countries are mainly suffering from political instability and armed, ethnic, or religious conflicts; thus, they are among the worst countries across the world, with high maternal mortality rates. According to the WHO (8), the Middle East and North African countries record 57 deaths per 100,000 livebirths on average, and United Arab Emirates records 3 deaths per 100,000 livebirths. Moreover, countries that have fragile situations and are conflict-affected, record 568-fold deaths per 100,000 births on average. This implies that women in war-affected countries are nearly 190 times more at risk of death during parturition as compared to UAE mothers. This situation is more serious in South Sudan, with the highest rate of maternal mortality (1,150 deaths per 100,000 livebirths). Chad stood at 1,140 deaths, and Sierra Leone recorded 1,120 deaths per 100,000 livebirths. As these countries are experiencing high maternal mortality rates, they have implemented plans to reduce the maternal mortality rate to below 70 deaths per 100,000 livebirth by 2030; however, the dilemma of high maternal mortality in Africa remains a problem.

Many researchers collectively agreed that maternal mortality is the hidden face of conflicts (9). Social insecurity, devastated healthcare infrastructures, poor health services, and lower female education are the main consequences of warfare on maternal care (3). Then the purpose of this study is to investigate the regional effects of political risks of conflicts on maternal mortality in the Middle East and Africa. Also, this study makes two contributions to the literature. First, this study is exhaustive in its coverage of the majority of socioeconomic and conflicts data sets which affect maternal mortality. Second, this study may indeed pioneer the use of a spatial panel data method for exploring the impacts of regional conflicts on maternal mortality in the Middle East and African countries. In this context, many researchers hold that the destructive effects of war permeate to neighboring countries. The existence of a strong economic, political, cultural, religious, and linguistic coherence among these countries leads to the expansion of such adverse events in a single country into a contagious process penetrating the neighboring countries. The transmission mechanism of the 2011 Arab Spring is an obvious example of the spatial effects of events in the region (10). Therefore, the prominent distinction of the present study is the consideration of the spatial features of the maternal mortality rate in the region.

## Literature Review

Warfare has adverse economic, social, political, and environmental consequences, resulting in poor health status in war-torn countries (11). Conflicts do not have the same effects on maternal mortality in different countries, and the scale of consequence depends on health infrastructures in the Pre-war period and governmental policies and priorities; however, channels through which warfare could affect maternal mortality are common in affected countries.

Political risks of conflicts impose adverse effects on maternal health through different channels, including undernourishment, famines, food shortages, which are the consequences of the eradication of croplands through warfare and put women in danger during pregnancies and after childbirth (12). The virement of healthcare budget to military expenditures, the destruction of health infrastructures, the shortage of skilled health professionals, and the rebate of medical staff would suspend obstetrical and maternal care in the long run and increase the risk of perilous miscarriage and pregnancy termination. Also, impoverishment, misery, and early marriage are the consequences of warfare, which stimulates maternal mortality (13). In addition, in most conflicts, the parties of war cut off all resources, block the ways of delivering health care and medical equipment to war-torn regions, and ban the mobility of emergency vehicles; the pregnant mothers could hardly have access to maternity care (14).

Refugee crises are another channel through which conflicts increase the maternal mortality rate due to the absence of emergency obstetric care, a large risk of infectious diseases spreading among pregnant, and higher malnutrition levels during pregnancies and after birth due to family economic crises in refugee shelters (3). The hygienic condition is poor in refugee camps, and the refugees struggle with a weak recycling and sanitation system, overcrowding, and a lack of access to clean water and food in camps. Refugee crises in the Middle East and Africa are an important issue. For example, the civil war in Burundi led to 300,000 deaths and a flow of millions of displaced people to the neighboring countries, e.g., Tanzania and the Democratic Republic of Congo. In northern Uganda, two million people have been displaced internally. According to UNHCR (15), Libya witnessed 226,000 internally displaced people, and there were 42,000 refugees. Also, 300,000 Palestinian refugees attempted to reside in Lebanon, and five million Syrians have been sheltered in the neighboring countries (such as Lebanon, Turkey, and Jordan). Refugees impose significant pressure on the host nations in order to meet their essential needs. Toole (16) reports that the maternal mortality rates in refugee camps were 100 times higher than the normal mortality rate. This is more critical to pregnant women who live outside camps and have no access to public health services or international aid. The adverse impacts of war on maternal mortality are not limited to war-affected countries; it has also spill-over effects on maternal health indicators in the neighboring/host countries.

There is a bulk of studies investigating the adverse health effects of war on life expectancy by Woldemicael (17), and Iqbal and Zorn (18), prevalence of contagious diseases by Gustafson et al. (19), disability-adjusted life years by Ghobarah et al. (20), infant mortality by and Wigley (21), maternal and child health by and Welander et al. (22), and gender gap in life expectancy by Neumayer and Plümper (2). Moreover, few studies in the literature covered the relationship between maternal mortality and conflicts. Mirzazada et al. (23) investigated the impacts of conflicts on maternal mortality in Afghanistan and compared various provinces based on the severity of the conflict through the Delphi process. They demonstrated that maternal health indicators were significantly lower in severe conflict provinces as compared to limited conflict provinces. They also mentioned that unfavorable economic and cultural conditions exacerbated the maternal mortality rate in war-affected provinces. Kotsadam and Ostby (24) employed the sisterhood method to investigate the effect of armed conflicts on the maternal mortality rates in thirty countries within Sub-Saharan Africa from 1989 to 2013. They reported that local conflicts increased the risk of maternal mortality, and this risk was different in rural areas from that in rich and more educated areas. Wagner et al. (25) analyzed the population effects of armed conflicts on Non-combatant vulnerable populations, especially on pregnant women ages 15–49 and orphanhood among children younger than 15 years of age in thirty-five African countries. They found that armed conflicts increased the risk of maternal deaths in Africa. Namasivayam (26) explored the effects of armed conflicts on the utilization of maternal health services in Uganda. They concluded that the utilization of contraception and institutional deliveries among women was significantly lower in northern Uganda as a conflict-affected region than in the rest of the country. Urdal and Che (3) examined the impacts of armed conflicts on fertility and maternal mortality across the world during 1970–2005. They concluded that the relationship between conflicts and the fertility rate was more significant in low-income countries and that the maternal mortality rates were very large in conflict-affected countries. Gopalan et al. (27) showed that maternal health in Asia and the Middle East is very weak and fragile in conflict-affected situations. Asi and Williams (28) showed that war-turned and conflict countries are more risky places to pregnant mothers and they argue that applying digital health systems may reduce maternal mortality rate and make progress in achieving sustainable development goals. El-Kak et al. (29) showed Syrian refugees crisis (resulting from war) and the increasing rate of Syrian refugee concentration in Lebanon caused a high maternal mortality rate in Lebanon for the period of 2010–2018. Gopalan et al. (30) showed that acute large conflicts have significant effects on maternal care-seeking and usage in Egypt. Akseer et al. (4) showed that poor access to essential reproductive and maternal health services in conflict countries caused maternal and child mortality rates to remain consistently higher than Non-conflict countries since 1990. Rocanello-Snow (31) showed that incidences of terrorist attacks, conflict, war, and political instability in Afghanistan affected the utilization of maternal healthcare services and the maternal mortality ratio. Alhassan et al. (32) also investigated alternative ways in times of disasters and emergencies in order to control the mortality rate. They recommend telehealth which increases access to healthcare and increases in quality of life which in turn reduces costs and easy access to healthcare services. Chukwuma et al. (33) did not find a significant difference in the impact of violent and Non-violent conflicts on the process quality of care and they showed that in Kenya, deterioration of equipment and infrastructure does not appear to be the main mechanism through which conflict has affected maternal care quality.

The review of the literature indicated that the spatial feature of health problems that arise from the war in neighboring countries have been ignored in previous works. Thus, this paper seeks to fill this gap by applying the spatial econometrics approach.

## Methods

### Spatial Econometric Model

The present paper proposes an experimental model considering the socio-economic and political explanatory variables of maternal mortality in the Middle East and Africa in line with Wang (34) and Zolala et al. (35). According to Model 1, the logarithm of the maternal mortality per 100,000 livebirths (*lnMATE*_*it*_) is a function of some explanatory variables, including the logarithm of GDP per capita (*lnGDP*), trade openness (*lnOPE*), natural resources rents (*lnRENT*), urbanization (*lnURB*), and conflicts (such as internal and external conflicts (*lnCON*)). The linear form of Equation (1) is used for experimental estimation:


(1) $$\begin{array}{l} lnMATE_{it}=\beta_{1}+\beta_{2}\;lnGDP_{it}+\beta_{3}\;lnOPE_{it}+\beta_{4}\;lnRENT_{it}+\\ \;\beta_{5}\;lnURB_{it}+\beta_{6}\;lnCON_{it}+c_{i}\left(optional\right)+\\ \;\alpha_{t}\left(optional\right)+\upsilon_{it} \end{array}$$

Where *c*_*i*_ is a spatial specific effect, and α_*t*_ represents a time-period specific effect. A few variables, such as urbanization, energy consumption, and trade openness, are generally used as explanatory variables in the literature on maternal mortality rates (34–36). Previous works stated that the share of health budget could extend when GDP has risen and economic development has happened in the region; therefore, the country could experience more trained health staff, provide more desirable primary health care, improve pregnant mothers' skills and knowledge to help reduce the maternal mortality rate. Also, urbanization is an important indicator of maternal mortality since it could improve infrastructures such as drinking water delivery, a strong disposal system, further hospitals, and ambulances. It could also improve women's education and training and provide strong maternal care, which could decrease the maternal mortality rate in urban areas (9). The effect of natural resource rents on the maternal mortality rate is vague. In some cases, natural resource-endowed countries could efficiently allocate resource rents to health expenditure and educate people. This would help decrease maternal mortality; otherwise, the resource rent converts into a curse of resources (37).

The spillover effects of higher internal and external conflicts on maternal mortality can be positive or negative, depending on the impacts of internal and external conflict on the financial resources allocated to health services. Thus, to separate the effects of the economic and Non-economic dimensions of conflicts on maternal mortality rates, the interaction terms of conflicts and GDP per capita are incorporated in a new form as:


(2) $$\begin{array}{l} \;lnMATE_{it}=\beta_{1}+\beta_{2}\;lnGDP_{it}+\beta_{3}\;lnOPE_{it}+\\ \beta_{4}\;lnRENT_{it}+\beta_{5}\;lnURB_{it}+\beta_{6}\;lnCON_{it}+\\ \beta_{7}\left(\;lnGDP_{it}\times lnCON_{it}\right)+c_{i}\left(optional\right)+\\ \alpha_{t}\left(optional\right)+\upsilon_{it} \end{array}$$

where ( *lnGDP*_*it*_×*lnCON*_*it*_) denotes the interaction term, while the coefficient of ( *lnGDP*_*it*_×*lnCON*_*it*_) represents the interaction between the national conflict and GDP per capita. The effect of GDP per capita on maternal mortality is formulated as:


(3) $$\begin{array}{l} \frac{d\left(\;lnMATE_{it}\right)}{d\left(\;lnGDP_{it}\right)}=\beta_{2}+\beta_{7}\;lnCON_{it} \end{array}$$

where β_7_ is a positive coefficient, suggesting that the uncertainty arising from different types of conflicts leads to lower governmental financial resources allocated to the healthcare system, and vice versa. While coefficient β_6_ shows the direct and Non-economic effects of conflicts on the rate of maternal mortality.

The present study explores the impacts of maternal mortality factors and emphasizes the indicators of both internal and external conflicts to shed light on the spatial relationships between observations. To this end, several spatial models were incorporated. Anselin et al. (38) argued that a spatial panel model could incorporate a lagged dependent parameter or apply a spatial autoregressive procedure into the error term. Furthermore, the spatial Durbin model was proposed by LeSage and Pace (39). The spatial Durbin model involves spatially-lagged independent variables. The mathematical formulations of the spatial lag (4), spatial error (5), and spatial Durbin (6) models are represented as:


(4) $$\begin{array}{l} y_{it}=\lambda \sum_{j\;=\;1}^{N}w_{ij}y_{jt}+\varphi +x_{it}\beta +c_{i}\left(optional\right)+\\ \alpha_{t}\left(optional\right)+\upsilon_{it} \end{array}$$

(5) $$\begin{array}{l} y_{it}=\lambda \sum_{j\;=\;1}^{N}w_{ij}y_{jt}+\varphi +x_{it}\beta +\sum_{j\;=\;1}^{N}w_{ij}x_{ijt}\theta +c_{i}\left(optional\right)+\\ \alpha_{t}\left(optional\right)+\upsilon_{it} \end{array}$$

(6) $$\begin{array}{l} y_{it}=\lambda \sum_{j\;=\;1}^{N}w_{ij}y_{jt}+\varphi +x_{it}\beta +\sum_{j\;=\;1}^{N}w_{ij}x_{ijt}\theta +c_{i}\left(optional\right)+\\ \alpha_{t}\left(optional\right)+\upsilon_{it} \end{array}$$

in which *y*_*it*_ denotes a dependent variable for cross-sectional unit *i* (*i* = 1, 2, … *N*) at time *t* (*t* = 1, 2, …, *T*). *x*_*it*_ represents the 1 × K exogenous variable vector, β stands for a *K*×1 parameter vector, $\overset{N}{\underset{j\;=\;1}{\sum }}w_{ij}y_{jt}$ accounts for the interaction effect of the dependent variable *y*_*jt*_ in the neighboring units on the dependent variable, *w*_*ij*_ denotes element *i, j* of the Pre-defined Non-negative *N*×*N* matrix of spatial weights, λ stands for the responses of the endogenous interaction effects, υ_*it*_ represents the error term with independent and identical distribution.

### Data and Summary Statistics

The data of forty-six countries in Africa and the Middle East from 2001 to 2017 were collected to investigate the effects of maternal mortality determinants. A summary of the constructed variables used in the analysis is presented in Table 1. Also, Table 2 reports the summarized statistics. All variables are presented in the logarithmic form; therefore, the estimated coefficients are elasticity.

**Table 1:** Variables used in the study.

**Variable**	**Variable constructed**	**Source**
*lnMATE*_*it*_	*lnMATE*_*it*_ = log(*MATE*_*it*_) *MATE*_*it*_= Maternal mortality ratio (per 100,000 livebirths) in the country *i* in period *t*	WDI
*lnGDP*_*it*_	*lnGDPP*_*it*_ = log(*GDPP*_*it*_) *GDP*_*it*_= GDP per capita in 2010 prices$	WDI
*lnOPE*_*it*_	*lnOPE*_*it*_ = log(*OPE*_*it*_) *OPE*_*it*_= Trade Openness (total exports and imports as a percentage of GDP)	WDI
*lnRENT*_*it*_	*lnRENT*_*it*_ = log(*RENT*_*it*_) *RENT*_*it*_= Total natural resources rents (as a percentage of GDP)	WDI
*lnURB*_*it*_	*lnURB*_*it*_ = log(*URB*_*it*_) *URB*_*it*_= Urban population (as a percentage of the total population)	WDI
*lnWAI*_*it*_	*lnWAI*_*it*_ = *log*(1 + 10 × *WAI*_*it*_) *WAI*_*it*_= War Index	PRS
*lnCCI*_*it*_	*lnCCI*_*it*_ = *log*(1 + 10 × *CBI*_*it*_) *CCI*_*it*_= Cross-Border Conflict Index	PRS
*lnFPI*_*it*_	*lnFPI*_*it*_ = *log*(1 + 10 × *FPI*_*it*_) *FPI*_*it*_= Foreign Pressures Index	PRS
*lnCWI*_*it*_	*lnCWI*_*it*_ = *log*(1 + 10 × *CWI*_*it*_) *CWI*_*it*_= Civil War Index	PRS
*lnTEI*_*it*_	*lnTEI*_*it*_ = *log*(1 + 10 × *TEI*_*it*_) *TEI*_*it*_= Terrorism Index	PRS
*lnCDI*_*it*_	*lnCDI*_*it*_ = *log*(1 + 10 × *CDI*_*it*_) *CDI*_*it*_= Civil Disorder Index	PRS

*WDI, World Development Indicator; https://datacatalog.worldbank.org/dataset/world-development-indicators.*

*PRS, Political Risk Services International Country Risk Guide; www.prsgroup.com*.

**Table 2:** Summary of statistics during 2001–2017.

	***lnMATE*_*it*_**	***lnGDP*_*it*_**	***lnOPE*_*it*_**	***lnRENT*_*it*_**	***lnURB*_*it*_**	***lnWAI*_*it*_**	***lnCCI*_*it*_**	***lnFPI*_*it*_**	***lnCWI*_*it*_**	***lnTEI*_*it*_**	***lnCDI*_*it*_**
Mean	5.21	7.77	4.26	6.60	3.88	3.65	3.43	3.30	3.52	3.23	3.24
Median	5.88	7.49	4.25	6.96	3.89	3.71	3.43	3.43	3.58	3.26	3.26
Max	7.72	11.15	5.79	8.84	4.61	3.71	3.71	3.71	3.71	3.71	3.71
Min	1.10	5.61	2.95	0.00	2.69	2.77	0.00	0.00	0.00	0.00	1.92
Std.D	1.54	1.40	0.46	1.88	0.48	0.12	0.30	0.44	0.29	0.36	0.21
Obs	782	782	782	782	782	782	782	782	782	782	782

**Figure 1:**
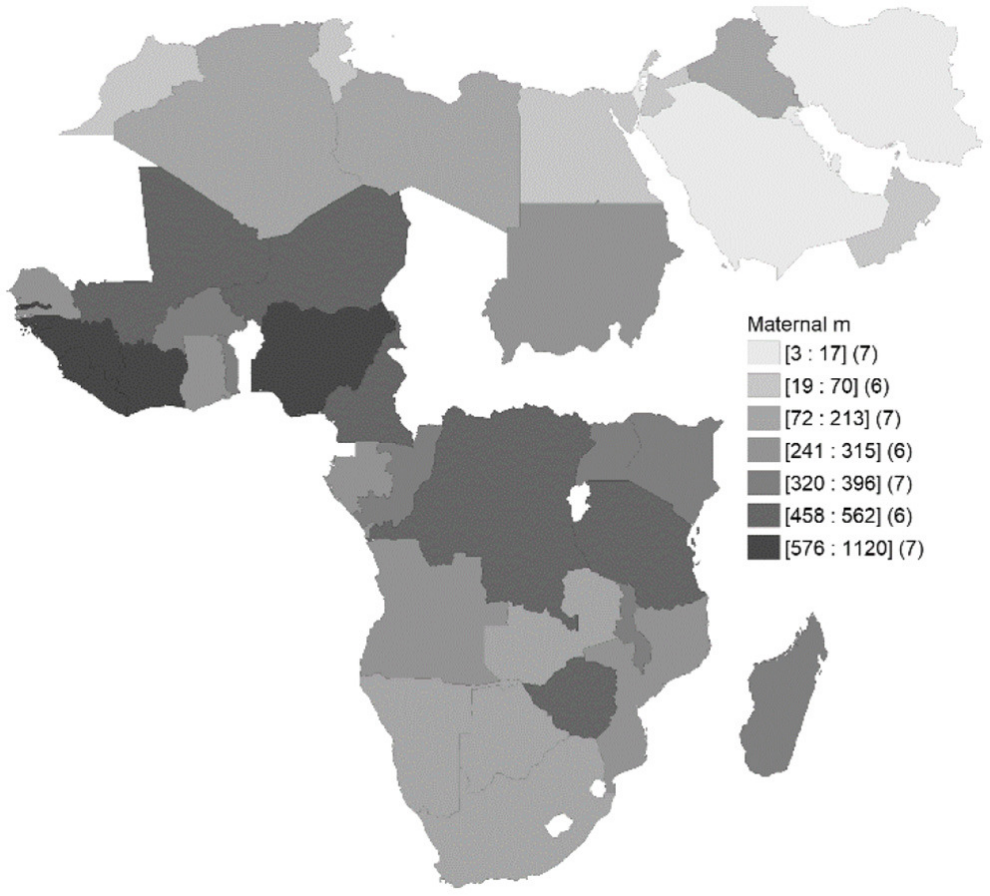
Maternal mortality (per 100,000 livebirths) across select African countries, (2017).

Figure 1 shows spatial distribution of maternal mortality (per 100,000 livebirths) across countries. It illuminates the spatial interactions between different countries and the regional integration in maternal mortality.

**Figure 2:**
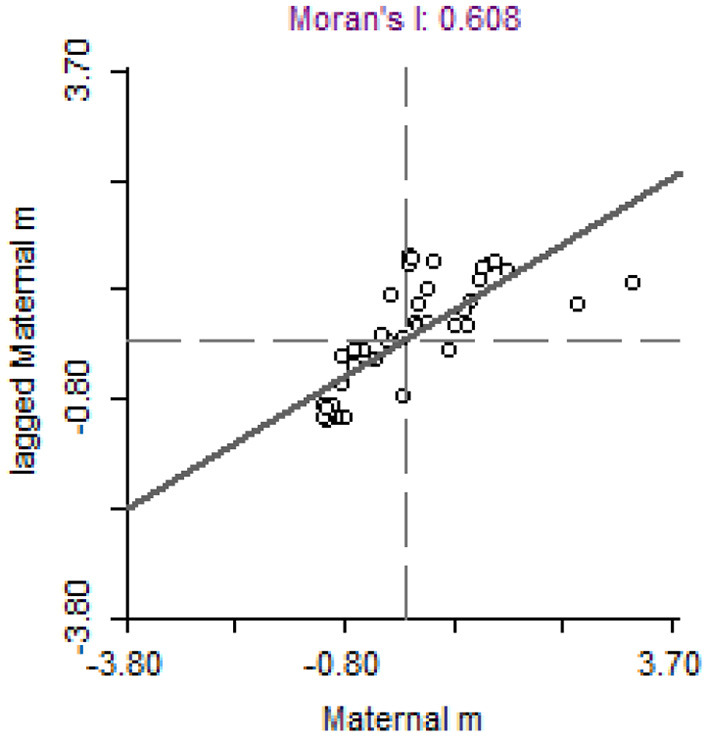
Moran's I statistics across countries.

To accurately demonstrate the spatial effects, Moran's I test statistics is provided in Figure 2. The regional observations and their spatial lag data are the dimensions of Figure 2. A positive Moran's I represent the spatial accumulation of similar values around the region, while a negative value represents the spatial accumulation of Non-similar values. Most countries exhibit a positive autocorrelation; as can be inferred from the fitting lines, the positive autocorrelation is dominant. Moran's I statistics indicate that the neighboring countries have more similar maternal mortality rates. Therefore, spatial econometric models are employed to investigate determinants of maternal mortality.

## Results

To investigate the probability of the time-period fixed effects and spatial fixed effects in the model, two separate likelihood ratio (LR) tests were used. For this purpose, the model with simultaneous spatial and time-period fixed effects is compared to the model of time-period fixed effects and/or the model of spatial fixed effects. If the null hypothesis is rejected, the model with simultaneous spatial and time-period fixed effects is selected. On the other hand, if the null hypothesis is accepted, the subsequent model is selected. LR test statistics for the models are presented in Table 3. The test results indicate the significance of the LR test statistics and the rejection of the null hypothesis for only the time-period fixed effects in most models. Therefore, the spatial fixed effects are selected as the best model to proceed with estimation.

**Table 3:** Spatial lag or the spatial error in the spatial and time-period fixed-effect model.

		**Pooled OLS**		**Spatial fixed effects**		**Time-period fixed effects**		**Spatial and time-period fixed effects**	
Model 1	LM spatial lag	505.04	(0.000^***^)	510.981	(0.000^***^)	436.341	(0.000^***^)	14.181	(0.000^***^)
	LM spatial error	426.882	(0.000^***^)	134.905	(0.000^***^)	245.647	(0.000^***^)	23.029	(0.000^***^)
	LR-test			850.359	(0.000^***^)	2,579.152	(0.000^***^)		
Model 2	LM spatial lag	478.951	(0.000^***^)	514.32	(0.000^***^)	402.464	(0.000^***^)	14.229	(0.000^***^)
	LM spatial error	433.262	(0.000^***^)	133.092	(0.000^***^)	231.314	(0.000^***^)	23.101	(0.000^***^)
	LR-test			850.349	(0.000^***^)	2,534.489	(0.000^***^)		
Model 3	LM spatial lag	502.697	(0.000^***^)	512.897	(0.000^***^)	428.318	(0.000^***^)	13.583	(0.000^***^)
	LM spatial error	423.204	(0.000^***^)	134.733	(0.000^***^)	235.993	(0.000^***^)	20.127	(0.000^***^)
	LR-test			864.17	(0.000^***^)	2,588.031	(0.000^***^)		
Model 4	LM spatial lag	491.327	(0.000^***^)	505.57	(0.000^***^)	413.436	(0.000^***^)	12.505	(0.000^***^)
	LM spatial error	413.805	(0.000^***^)	127.38	(0.000^***^)	222.655	(0.000^***^)	18.648	(0.000^***^)
	LR-test			855.947	(0.000^***^)	2,571.463	(0.000^***^)		
Model 5	LM spatial lag	519.168	(0.000^***^)	511.812	(0.000^***^)	448.245	(0.000^***^)	13.72	(0.000^***^)
	LM spatial error	425.409	(0.000^***^)	133.778	(0.000^***^)	244.191	(0.000^***^)	21.89	(0.000^***^)
	LR-test			849.71	(0.000^***^)	2,580.347	(0.000^***^)		
Model 6	LM spatial lag	466.189	(0.000^***^)	511.201	(0.000^***^)	394.267	(0.000^***^)	13.953	(0.000^***^)
	LM spatial error	369.173	(0.000^***^)	133.594	(0.000^***^)	183.685	(0.000^***^)	22.636	(0.000^***^)
	LR-test			848.461	(0.000^***^)	2,544.418	(0.000^***^)		
Model 7	LM spatial lag	505.81	(0.000^***^)	481.06	(0.000^***^)	440.957	(0.000^***^)	12.184	(0.000^***^)
	LM spatial error	428.952	(0.000^***^)	105.453	(0.000^***^)	248.925	(0.000^***^)	18.046	(0.000^***^)
	LR-test			813.734	(0.000^***^)	2,577.738	(0.000^***^)		
Model 8	LM spatial lag	470.9	(0.000^***^)	499.883	(0.000^***^)	402.605	(0.000^***^)	13.253	(0.000^***^)
	LM spatial error	410.133	(0.000^***^)	111.062	(0.000^***^)	224.7	(0.000^***^)	20.895	(0.000^***^)
	LR-test			826.121	(0.000^***^)	2,549.348	(0.000^***^)		
Model 9	LM spatial lag	490.371	(0.000^***^)	506.781	(0.000^***^)	425.25	(0.000^***^)	13.343	(0.000^***^)
	LM spatial error	391.695	(0.000^***^)	130.434	(0.000^***^)	223.661	(0.000^***^)	19.573	(0.000^***^)
	LR-test			853.453	(0.000^***^)	2,588.222	(0.000^***^)		
Model 10	LM spatial lag	491.745	(0.000^***^)	509.193	(0.000^***^)	418.404	(0.000^***^)	11.981	(0.001^***^)
	LM spatial error	403.248	(0.000^***^)	129.037	(0.000^***^)	220.482	(0.000^***^)	17.775	(0.000^***^)
	LR-test			857.485	(0.000^***^)	2,574.464	(0.000^***^)		
Model 11	LM spatial lag	519.775	(0.000^***^)	475.895	(0.000^***^)	458.1	(0.000^***^)	13.493	(0.000^***^)
	LM spatial error	419.215	(0.000^***^)	93.856	(0.000^***^)	252.215	(0.000^***^)	21.495	(0.000^***^)
	LR-test			825.83	(0.000^***^)	2,581.065	(0.000^***^)		
Model 12	LM spatial lag	469.207	(0.000^***^)	497.582	(0.000^***^)	402.466	(0.000^***^)	13.893	(0.000^***^)
	LM spatial error	369.593	(0.000^***^)	116.908	(0.000^***^)	191.116	(0.000^***^)	22.477	(0.000^***^)
	LR-test			836.458	(0.000^***^)	2,544.17	(0.000^***^)		
Model 13	LM spatial lag	529.465	(0.000^***^)	436.868	(0.000^***^)	473.684	(0.000^***^)	12.188	(0.000^***^)
	LM spatial error	470.308	(0.000^***^)	58.735	(0.000^***^)	291.87	(0.000^***^)	18.045	(0.000^***^)
	LR-test			775.209	(0.000^***^)	2,571.096	(0.000^***^)		

p-value, ^***^ show significance at 1% level.

*Authors' estimations*.

Another test in Table 3 is used to examine whether the inclusion of the spatial lag or spatial error in the model in the absence of spatial interaction effects would lead to a significant improvement in the model or not. Thus, using the residuals of a Non-spatial model, the Lagrange Multiplier (LM) statistics tests are applied to a spatially-lagged dependent variable and spatial error autoregressive (40). The test statistic has the chi-square distribution. If the null hypothesis of the LM test is rejected, the presence of the spatial lagged model and the spatial error model is confirmed. Since the LR test results confirmed the existence of the spatial fixed effects, this study examines only LM statistics for this model.

The results presented in Table 3 suggest that the test statistic values in all models are significant at the level of one percent. Therefore, spatial lagged and spatial error effects must be considered in the maternal mortality model. Hence, the presence of spatial interaction effects in the model emphasizes the need to consider such effects when investigating the factors affecting maternal mortality in experimental studies.

Table 4 presents the Hausman test results to examine the possibility of replacing the fixed-effect model with a random-effect model. The null hypothesis in this test emphasizes the existence of random effects in the model. The results of the Hausman test reject the assumption of random effects in the spatial lag model for all the models and confirm the existence of fixed effects at a significance level of 1%.

**Table 4:** Spatial Durbin model and Hausman test results.

	**Hausman test**		**Wald test**		**LR test**	
	**Spatial Durbin model**	**Spatial lag model**	**Spatial Durbin model against spatial lag model**	**Spatial Durbin model against spatial error model**	**Spatial Durbin model against spatial lag model**	**spatial Durbin model against spatial error model**
Model 1	26.244	15.27	38.992	41.117	46.641	48.5
	(0.000***)	(0.084*)	(0.000***)	(0.000***)	(0.000***)	(0.000***)
Model 2	30.655	20.026	49.153	50.013	58.116	60.456
	(0.000***)	(0.045**)	(0.000***)	(0.000***)	(0.000***)	(0.000***)
Model 3	24.613	12.081	38.026	40.285	45.808	47.913
	(0.000***)	(0.358)	(0.000***)	(0.000***)	(0.000***)	(0.000***)
Model 4	23.532	14.917	57.057	58.173	66.735	67.914
	(0.001***)	(0.186)	(0.000***)	(0.000***)	(0.000***)	(0.000***)
Model 5	28.754	27.039	49.675	51.837	59.2	60.299
	(0.000***)	(0.005***)	(0.000***)	(0.000***)	(0.000***)	(0.000***)
Model 6	30.471	25.244	52.788	55.08	62.771	63.831
	(0.000***)	(0.008***)	(0.000***)	(0.000***)	(0.000***)	(0.000***)
Model 7	28.097	28.257	36.18	37.761	43.118	44.288
	(0.000***)	(0.003***)	(0.000***)	(0.000***)	(0.000***)	(0.000***)
Model 8	27.911	22.076	51.665	52.622	60.985	63.934
	(0.000***)	(0.054*)	(0.000***)	(0.000***)	(0.000***)	(0.000***)
Model 9	22.255	13.149	37.944	40.939	46.036	48.704
	(0.002***)	(0.436)	(0.000***)	(0.000***)	(0.000***)	(0.000***)
Model 10	26.268	14.508	63.135	64.048	73.391	75.575
	(0.000***)	(0.339)	(0.000***)	(0.000***)	(0.000***)	(0.000***)
Model 11	25.639	18.843	45.225	48.786	53.538	56.275
	(0.001***)	(0.128)	(0.000***)	(0.000***)	(0.000***)	(0.000***)
Model 12	37.304	30.431	83.386	83.868	91.134	93.201
	(0.000***)	(0.004***)	(0.000***)	(0.000***)	(0.000***)	(0.000***)
Model 13	35.36	21.347	56.304	56.18	60.632	61.725
	(0.000***)	(0.066*)	(0.000***)	(0.000***)	(0.000***)	(0.000***)

p-value, ^***^, ^**^, and ^*^ show significance at 1%, 5%, and 10% level, respectively.

*Authors' estimations*.

Finally, this study evaluates two separate hypotheses *H*_0_:θ = 0 and *H*_0_:θ + λβ= 0 in Equation (6). If the first hypothesis is the case, the spatial Durbin model is simplified into the spatial lag model. On the other hand, if the second hypothesis is the case, the spatial Durbin model can be simplified into a spatial error model (41). The present work uses the LR test or Wald test to explore whether the existence of the spatial lagged independent variable in the model is significant. Each of these two tests has positive and negative characteristics. LR tests are employed to estimate more models, while Wald tests are sensitive to nonlinear constraints.

The test results in Table 4 provide the same outcome for the fixed- and random-effect models. The statistical value of the two tests (i.e., the LR and the Wald test) is significant for all models, and the spatial Durbin model cannot be converted into the spatial error model and spatial lag model in any of the models. Therefore, the existence of the spatial lagged independent variable is also confirmed; finally, the spatial Durbin model is the basis for the analysis of the estimation results.

According to Table 5, an increase of 1% in the GDP per capita leads to a significant decrease of nearly 0.474% in the maternal mortality rate. However, trade openness does not show a significant effect. Also, the growth of natural resource rents in most models leads to a significant reduction in maternal mortality rates. Urbanization is one of the positive dimensions in the enhancement of maternal mortality rates in the countries under study. Among the conflict indicators, a rise in war and cross-border conflicts index (which shows war and cross border war decrease), raises the maternal mortality rate which is in contrast to theoretical implications. Whereas rise in other indexes (which shows a decrease in foreign pressure, civil war, and civil disorder), yield a lower mortality rate.

**Table 5:** Estimation results for Equation (1).

	**Model 1**	**Model 2**	**Model 3**	**Model 4**	**Model 5**	**Model 6**	**Model 7**
*lnGDP*	−0.474	−0.473	−0.469	−0.467	−0.433	−0.466	−0.464
	(0.000***)	(0.000***)	(0.000***)	(0.000***)	(0.000***)	(0.000***)	(0.000***)
*lnOPE*	0.005	−0.001	0.007	−0.005	0.005	0.008	0.004
	(0.786)	(0.977)	(0.741)	(0.788)	(0.783)	(0.689)	(0.858)
*lnRENT*	−0.022	−0.014	−0.02	−0.017	−0.021	−0.02	−0.021
	(0.018**)	(0.124)	(0.028**)	(0.054*)	(0.016**)	(0.027**)	(0.019**)
*lnURB*	0.284	0.345	0.312	0.242	0.227	0.267	0.294
	(0.008***)	(0.001***)	(0.003***)	(0.023**)	(0.029**)	(0.011**)	(0.006***)
*lnWAR*		0.205					
		(0.000***)					
*lnCCI*			0.037				
			(0.000***)				
*lnFPI*				−0.036			
				(0.000***)			
*lnCWI*					−0.077		
					(0.000***)		
*lnTEI*						−0.037	
						(0.000***)	
*lnCDI*							−0.062
							(0.004***)
*W*×*lnGDP*	−0.212	−0.199	−0.193	−0.283	−0.228	−0.239	−0.178
	(0.044**)	(0.056*)	(0.064*)	(0.007***)	(0.034**)	(0.022**)	(0.099*)
*W*×*lnOPE*	0.015	0.012	0.014	−0.051	0.041	0.04	0.031
	(0.817)	(0.855)	(0.828)	(0.427)	(0.514)	(0.535)	(0.635)
*W*×*lnRENT*	0.092	0.089	0.086	0.123	0.107	0.092	0.089
	(0.000***)	(0.000***)	(0.000***)	(0.000***)	(0.000***)	(0.000***)	(0.000***)
*W*×*lnURB*	−0.75	−0.679	−0.773	−0.877	−0.755	−0.698	−0.718
	(0.000***)	(0.001***)	(0.000***)	(0.000***)	(0.000***)	(0.000***)	(0.000***)
*W*×*lnWAR*		−0.472					
		(0.008***)					
*W*×*lnCCI*			−0.017				
			(0.618)				
*W*×*lnFPI*				−0.092			
				(0.000***)			
*W*×*lnCWI*					−0.051		
					(0.174)		
*W*×*lnTEI*						−0.085	
						(0.000***)	
*W*×*lnCDI*							−0.034
							(0.544)
*W*×*lnMATE*	0.018	0.037	0.035	−0.074	−0.038	−0.06	0.003
	(0.788)	(0.572)	(0.599)	(0.287)	(0.579)	(0.39)	(0.969)

p-value, ^***^, ^**^, and ^*^ show significance at 1%, 5%, and 10% level respectively.

*Authors' estimations*.

Accordingly, Table 6 examines the spillover effects of conflicts and also provides the estimation results of Equation (5). The incorporation of the spillover effects of internal and external conflicts on GDP per capita yielded the same and significant results for all types of conflicts. The Non-inclusion of the interaction term seems to produce misleading results. Placing the estimated coefficients in Equation (6), the spillover effects are negative for some conflict variables and positive for some others:


(7) $$\begin{array}{l} \frac{d\left(\;lnMATE_{it}\right)}{d\left(\;lnGDP_{it}\right)}=-1.514\;+\;0.172\times lnWAR_{it} \end{array}$$

(8) $$\begin{array}{l} \frac{d\left(\;lnMATE_{it}\right)}{d\left(\;lnGDP_{it}\right)}=-0.693\;+\;0.036\times lnCCI_{it} \end{array}$$

(9) $$\begin{array}{l} \frac{d\left(\;lnMATE_{it}\right)}{d\left(\;lnGDP_{it}\right)}=-0.18-0.046\times lnFPI_{it} \end{array}$$

(10) $$\begin{array}{l} \frac{d\left(\;lnMATE_{it}\right)}{d\left(\;lnGDP_{it}\right)}=-0.149-0.096\times lnCWI_{it} \end{array}$$

(11) $$\begin{array}{l} \frac{d\left(\;lnMATE_{it}\right)}{d\left(\;lnGDP_{it}\right)}=-0.226-0.042\times lnTEI_{it} \end{array}$$

(12) $$\begin{array}{l} \frac{d\left(\;lnMATE_{it}\right)}{d\left(\;lnGDP_{it}\right)}=-0.003-0.074\times lnCDI_{it} \end{array}$$

**Table 6:** Estimation results for Equation (6).

	**Model 8**	**Model 9**	**Model 10**	**Model 11**	**Model 12**	**Model 13**
*lnGDP*	−1.514	−0.693	−0.18	0.149	−0.226	−0.003
	(0.000***)	(0.000***)	(0.017)	(0.183)	(0.011)	(0.981)
*lnOPE*	0.009	0.014	0.002	−0.007	0.002	0.005
	(0.653)	(0.472)	(0.936)	(0.709)	(0.904)	(0.809)
*lnRENT*	−0.011	−0.021	−0.016	−0.014	−0.014	−0.017
	(0.208)	(0.02**)	(0.069*)	(0.115)	(0.126)	(0.058*)
*lnURB*	0.307	0.29	0.311	0.299	0.322	0.249
	(0.003***)	(0.006***)	(0.003***)	(0.003***)	(0.002***)	(0.017)
*lnWAR*	−1.152					
	(0.000***)					
*lnGDP*×*lnWAR*	0.172					
	(0.000***)					
*lnCCI*		−0.24				
		(0.002***)				
*lnGDP*×*lnCCI*		0.036				
		(0.000***)				
*lnFPI*			0.328			
			(0.000***)			
*lnGDP*×*lnFPI*			−0.046			
			(0.000***)			
*lnCWI*				0.636		
				(0.000***)		
*lnGDP*×*lnCWI*				−0.096		
				(0.000***)		
*lnTEI*					0.299	
					(0.01**)	
*lnGDP*×*lnTEI*					−0.042	
					(0.003***)	
*lnCDI*						0.5
						(0.000***)
*lnGDP*×*lnCDI*						−0.074
						(0.000***)
*W*×*lnGDP*	0.973	−0.092	−0.588	−0.749	−1.32	−1.386
	(0.141)	(0.705)	(0.004***)	(0.057*)	(0.000***)	(0.000***)
*W*×*lnOPE*	0.009	0.006	−0.042	0.074	0.051	0.027
	(0.886)	(0.922)	(0.509)	(0.238)	(0.415)	(0.66)
*W*×*lnRENT*	0.084	0.088	0.119	0.089	0.063	0.062
	(0.000***)	(0.000***)	(0.000***)	(0.000***)	(0.007***)	(0.009***)
*W*×*lnURB*	−0.664	−0.704	−0.956	−0.747	−0.859	−0.832
	(0.001***)	(0.000***)	(0.000***)	(0.000***)	(0.000***)	(0.000***)
*W*×*lnWAR*	1.072					
	(0.198)					
*W*×*lnGDP*×*lnWAR*	−0.189					
	(0.069*)					
*W*×*lnCCI*		0.142				
		(0.535)				
*W*×*lnGDP*×*lnCCI*		−0.021				
		(0.478)				
*W*×*lnFPI*			−0.489			
			(0.062*)			
*W*×*lnGDP*×*lnFPI*			0.052			
			(0.122)			
*W*×*lnCWI*				−0.684		
				(0.126)		
*W*×*lnGDP*×*lnCWI*				0.085		
				(0.159)		
*W*×*lnTEI*					−1.584	
					(0.000***)	
*W*×*lnGDP*×*lnTEI*					0.182	
					(0.000***)	
*W*×*lnCDI*						−1.404
						(0.000***)
*W*×*lnGDP*×*lnCDI*						0.19
						(0.000***)
*W*×*lnMATE*	0.069	0.036	−0.061	0.004	−0.125	0.002
	(0.291)	(0.589)	(0.381)	(0.956)	(0.078)	(0.977)

p-value, ^***^, ^**^, and ^*^ show significance at 1%, 5%, and 10% level, respectively.

*Authors' estimations*.

The spatial models allow for separating the direct and indirect effects of dependent variables. Direct effects measure the effects of independent variables on the dependent variable of a given country, whereas spillover effects measure the effects of independent variables in the neighboring countries on the dependent variable of a given country. Table 7 reports the direct and indirect effects of all variables for Model 1 and the variables of the internal and external conflicts for Models 2–7. A comparison of Tables 6, 7 suggests that the direct effects are slightly different from the estimates since the indirect effects include feedback effects resulting from the effects of crossing neighboring states and returning to the state of origin.

**Table 7:** Marginal effects of the maternal mortality determinants.

	**Direct**		**Indirect**		**Total**	
	**Coefficient**	***p*-value**	**Coefficient**	***p*-value**	**Coefficient**	***p*-value**
*lnGDPP*	−0.476	(0.000***)	−0.218	(0.031**)	−0.694	(0.000***)
*lnOPE*	0.006	(0.777)	0.015	(0.819)	0.021	(0.765)
*lnRENT*	−0.022	(0.017**)	0.093	(0.000***)	0.071	(0.004***)
*lnURB*	0.282	(0.009***)	−0.756	(0.000***)	−0.475	(0.02**)
*lnWAR*	0.203	(0.000***)	−0.486	(0.011**)	−0.283	(0.159)
*lnCCI*	0.037	(0.001***)	−0.016	(0.656)	0.02	(0.596)
*lnFPI*	−0.035	(0.000***)	−0.084	(0.001***)	−0.119	(0.000***)
*lnCWI*	−0.077	(0.000***)	−0.047	(0.183)	−0.124	(0.002***)
*lnTEI*	−0.036	(0.001***)	−0.079	(0.001***)	−0.115	(0.000***)
*lnCDI*	−0.062	(0.007***)	−0.036	(0.54)	−0.098	(0.13)

p-value, ^***^ and ^**^ show significance at 1% and 5% level, respectively.

*Authors' estimations*.

We focus on the spillover effects in Table 7 to examine the effects of the independent variables of the neighboring countries on maternal mortality in the origin country. The coefficient of the logarithmic GDP per capita in the neighboring countries is −0.218, indicating that the economic growth of a neighboring country reduces the maternal mortality rate. Also, the coefficient of trade openness in neighboring countries is insignificant. The effects of urbanization and natural resource rents are significantly negative and positive, respectively. The effects of the conflict indexes, including war, foreign pressures, and terrorism in neighboring countries are negative and significant.

## Discussion

### Main Findings

The present study spatially explored the regional determinants of maternal mortality rate with a special emphasis on the contribution of political risks imposed by internal and external conflicts. Data from forty-six African and Middle Eastern countries from 2011 to 2017 were used to examine the regional impacts of internal and external conflicts on maternal mortality rates. Diagnostic tests confirmed the existence of spatial interactions among variables; thus, the spatial Durbin model was used to investigate the effects of independent model variables. Based on the results, economic growth provides a basis for reducing maternal mortality. This result is similar to the results of Nwankwo (42), and Maruthappu et al. (43). The creation of wider health infrastructures and the expansion of health services in the economy are among the contributions of economic growth to countries. This in turn can drastically reduce maternal mortality. Although trade openness can reduce maternal mortality through increased imports of technological healthcare goods, our results reveal that such effects are insignificant in the countries under study. This result is in line with the results of Barlow (44), and Olper et al. (45) where they conclude that trade openness has no significant impact on health status (especially maternal and childhood health) in low- and middle-income countries. Also, in line with the results of Nikzadian et al. (37), this study shows that natural resource rents are a significant variable in reducing mortality rates within the countries under study, in the sense that countries provide the necessary financial resources to cover health expenditures through revenues by the sales of natural resources. The existing realities concerning the countries under study show the weak production structure of these countries; the tax revenues of these countries in the manufacturing sector are very low, and their health expenditures would encounter serious challenges in the absence of natural resource rents.

Also, according to the results, the expansion of urbanization has led to an increase in the maternal mortality rate. Although this result is inconsistent with theoretical bases, it is following the results of Akseer et al. (9) and Bornemisza et al. (46). On one hand, they argue that the intensity and frequency of internal and external conflicts are more in urbanized areas rather than un-urbanized areas and urban areas face more insurgency and political unrests in comparison with later ones. In addition, as population density (and consequently pregnant women crowed) is higher in urban areas, then the large scale of pregnant mothers are encountering in the risk of maternal mortality during war conditions. On the other hand, they argue that urbanization mainly rises inequality and disparities including household income inequalities, and disparities in access to welfare and healthcare facilities, especially at the initial levels of development. Therefore, urbanization couldn't improve maternal health and it seems that the Middle East and African countries are no exceptions to this trend and this result highlights the importance of governmental policies in reducing inequality to control the maternal mortality rate in the mentioned region.

After examining the effects of model control variables, the effects of internal and external conflicts were evaluated in the form of two different models. The results for some variables are inconsistent with theoretical expectations; for example, war and cross-border conflicts were found to reduce maternal mortality rates. The health sector seems to be highly reliant on how internal and external disputes impact the generation and distribution of specialized financial resources. The findings suggest that The data show that war and cross-border conflict exacerbate the beneficial impact of economic expansion on maternal mortality. Therefore, as such conflicts deepen, health financial resource allocations and health services grow, and the economy reorients its output toward health services. The results are quite different for most internal conflict components including civil war, terrorism, and civil disorder, plus foreign pressures, and incidence of these kinds of conflicts worsen maternal mortality rate. These results are in accordance with the results of Mirzazada et al. (23), Ruiz Cantero et al. (47) Kotsadam and Ostby (24), Wagner et al. (25), and Gizelis and Cao (12). Indeed, when such disputes become more prevalent in nations, the structure of output changes in a way that harms health care. Overall, most conflict variables increase maternal mortality by reducing health services in the economy. According to the results, political risks imposed by war, foreign pressures, and terrorism in neighboring countries have spillover effects on the regional extensions of maternal mortality rates and decrease mortality rates in the country of origin. This result is in line with the results of Bendavid et al. (4), Wagner et al. (25), and Wagner et al. (48).

### Conclusion and Policy Implications

The topic of conflict spillover effects on maternal mortality rate has been widely accepted in this study. The results of this research provide fresh insight into the maternal mortality implications of regional violence. The study's practical consequences are as follows: internal conflicts seem to be more detrimental to maternal health than the prospect of foreign war in the Middle East and Africa. As a result, these countries must establish a country-specific framework for resolving internal disputes and political turmoil at the national level. Therefore, these countries must adopt a country-specific mechanism through which they can overcome the occurrence of internal conflicts and national political unrests. In addition, as our results confirm the existence of spatial effects of conflicts and significant spillover effects in the Middle East and African region, then, policymakers in this region are recommended to design effective, region-wide strategies to deal with any kind of internal and external conflicts. Also, it is wise for policymakers to improve political relationships and establish multilateral organizations against external interventions and internal conflicts in the region.

Although the contribution of external conflicts on maternal mortality is insignificant and negligible, however, the effects of internal conflicts on maternal mortality are significantly considerable. Then building the health system resilient and resolving any disruption in maternal care accessibility should be the health policy priorities in mentioned countries. Therefore, paying attention to establishing obstetrics and gynecology clinics in neighborhood/local wide should be preferred to nation-wide health programs, because mothers mainly lose access to healthcare delivery systems during internal physical violence inside a country. In addition, as conflicts debilitate economic growth and adversely affect women's wellbeing, then local and Non-governmental organizations (NGOs) in the Middle East and Africa are needed to support pregnant women and supply maternity services as educated and skilled obstetricians and midwives, covering the maternity costs and educate mothers.

Although the most important limitation of this research was the availability of formal data for many countries, however, further researches are necessary to accurately determine which kind of maternal care process including antenatal service, postnatal, or delivery service supply become limited and not accessible for women in the occurrence of internal conflicts, to plan wisely to overcome deficiencies.

## Data Availability Statement

Publicly available datasets were analyzed in this study. This data can be found here: https://data.worldbank.org/.

## Author Contributions

JM, SR, and AH: conceptualization and methodology. MK: software, validation, and formal analysis. JM, MK, SR, and AH: writing-original draft, writing-review and editing, and visualization. All authors contributed to the article and approved the submitted version.

## Conflict of Interest

The authors declare that the research was conducted in the absence of any commercial or financial relationships that could be construed as a potential conflict of interest.

## Publisher's Note

All claims expressed in this article are solely those of the authors and do not necessarily represent those of their affiliated organizations, or those of the publisher, the editors and the reviewers. Any product that may be evaluated in this article, or claim that may be made by its manufacturer, is not guaranteed or endorsed by the publisher.

## References

[1] Swatzyna RJ Pillai VK. The effects of disaster on women's reproductive health in developing countries. Glob J Health Sci. (2013) 5:106–13. 10.5539/gjhs.v5n4p106 23777727 PMC4776806

[2] Plümper T Neumayer E. The unequal burden of war: the effect of armed conflict on the gender gap in life expectancy. Int Organ. (2006) 60:723–54. 10.1017/S0020818306060231

[3] Urdal H Che CP. War and gender inequalities in health: the impact of armed conflict on fertility and maternal mortality. Int Interact. (2013) 39:489–510. 10.1080/03050629.2013.805133

[4] Bendavid E Boerma T Akseer N Langer A Malembaka EB Okiro EA. The effects of armed conflict on the health of women and children. Lancet. (2021) 397:522–32. 10.1016/S0140-6736(21)00131-8 33503456 PMC7612212

[5] Obermeyer Z Murray CJL Gakidou E. Fifty years of violent war deaths from Vietnam to Bosnia: analysis of data from world health survey programme. BMJ. (2008) 336:1482–6. 10.1136/bmj.a137 18566045 PMC2440905

[6] Hogan MC Foreman KJ Naghavi M Ahn SY Wang M Makela SM. Maternal mortality for 181 countries, 1980–2008: a systematic analysis of progress towards Millennium Development Goal 5. Lancet. (2010) 375:1609–23. 10.1016/S0140-6736(10)60518-1 20382417

[7] O'Hare BAM Southall DP. First do no harm: the impact of recent armed conflict on maternal and child health in Sub-Saharan Africa. J R Soc Med. (2007) 100:564–70. 10.1177/0141076807100012015 18065709 PMC2121626

[8] World Health Organization (WHO). World Health Statistics 2017: Monitoring Health for the SDGs, Sustainable Development Goals. World Health Organization (2017). Available online at: https://apps.who.int/iris/handle/10665/255336

[9] Akseer N Wright J Tasic H Everett K Scudder E Amsalu R. Women, children and adolescents in conflict countries: an assessment of inequalities in intervention coverage and survival. BMJ Glob Heal. (2020) 5:e002214. 10.1136/bmjgh-2019-002214 32133179 PMC7042600

[10] Boserup RA, Hazbun, W, Makdisi, K, Malmvig, H,. New Conflict Dynamics Between Regional Autonomy Intervention in the Middle East North Africa. (2017). Available online at: https://pure.diis.dk/ws/files/830699/2017_DIIS_New_Conflict_Dynamics_in_ the_Middle_East_and_North_Africa_web.pdf

[11] Iqbal Z. Health and human security: The public health impact of violent conflict. Int Stud Q. (2006) 50:631–49. 10.1111/j.1468-2478.2006.00417.x

[12] Gizelis T-I Cao X. Peacekeeping and Post-conflict Maternal Health. Typescript Univ Essex Penn State Univ (2016).

[13] Kottegoda S Samuel K Emmanuel S. Reproductive health concerns in six conflict-affected areas of Sri Lanka. Reprod Health Matters. (2008) 16:75–82. 10.1016/S0968-8080(08)31359-7 18513609

[14] Chandrasekhar S Gebreselassie T Jayaraman A. Maternal health care seeking behavior in a post-conflict HIPC: the case of rwanda. Popul Res Policy Rev. (2011) 30:25–41. 10.1007/s11113-010-9175-0

[15] United Nations High Commissioner for Refugees (UNHCR) (2017). Global Trends: Forced Displacement in 2016. Available online at: https://www.unhcr.org/5b27be547.pdf

[16] Toole MJ. Displaced persons and war. War Public Heal. (2009)197–214. 10.1093/acprof:oso/9780195311181.003.0013

[17] Woldemicael G. Recent fertility decline in Eritrea: is it a conflict-led transition? Demogr Res. (2008) 18:27–58. 10.4054/DemRes.2008.18.2

[18] IQBAL Z. War and the Health of Nations. Stanford University Press (2019).

[19] Gustafson P Gomes VF Vieira CS Jensen H Seng R Norberg R. Tuberculosis mortality during a civil war in Guinea-Bissau. J Am Med Assoc. (2001) 286:599–603. 10.1001/jama.286.5.599 11476664

[20] Ghobarah HA Huth P Russett B. Civil wars kill and maim people - long after the shooting stops. Am Polit Sci Rev. (2003) 97:189–202. 10.1017/S0003055403000613

[21] Wigley S Akkoyunlu-Wigley A. The impact of democracy and media freedom on under-5 mortality, 1961–2011. Soc Sci Med. (2017) 190:237–46. 10.1016/j.socscimed.2017.08.023 28869904

[22] Burroway R. Democracy and child health in developing countries. Int J Comp Sociol. (2016) 57:338–64. 10.1177/002071521667651425982869

[23] Mirzazada S Padhani ZA Jabeen S Fatima M Rizvi A Ansari U. Impact of conflict on maternal and child health service delivery: a country case study of Afghanistan. Confl Health. (2020) 14:1–13. 10.1186/s13031-020-00285-x 32536966 PMC7288441

[24] Kotsadam A Østby G. Armed conflict and maternal mortality: a micro-level analysis of sub-Saharan Africa, 1989–2013. Soc Sci Med. (2019) 239:112526. 10.1016/j.socscimed.2019.112526 31520880

[25] Wagner Z Heft-Neal S Wise PH Black RE Burke M Boerma T. Women and children living in areas of armed conflict in Africa: a geospatial analysis of mortality and orphanhood. Lancet Glob Heal. (2019) 7:e1622–31. 10.1016/S2214-109X(19)30407-3 31669039 PMC7024993

[26] Namasivayam A González PA Delgado RC Chi PC. The effect of armed conflict on the utilization of maternal health services in Uganda: a population-based Study. PLoS Curr. (2017) 9:25–32. 10.1371/currents.dis.557b987d6519d8c7c96f2006ed3c271a 29188138 PMC5693797

[27] Gopalan SS Das A Howard N. Maternal and neonatal service usage and determinants in fragile and conflict-affected situations: A systematic review of Asia and the Middle-East. BMC Womens Health. (2017) 17:1–12. 10.1186/s12905-017-0379-x 28298198 PMC5353776

[28] Asi YM Williams C. The role of digital health in making progress toward Sustainable Development Goal (SDG) 3 in conflict-affected populations. Int J Med Inform. (2018) 114:114–20. 10.1016/j.ijmedinf.2017.11.003 29126701

[29] El Kak F Harb H Daouk S Nassar A Kabakian-Khasholian T. Maternal mortality in Lebanon: increased vulnerability among Syrian refugees. Int J Gynecol Obstet. (2021) 1–7. 10.1002/ijgo.14063 34890470

[30] Gopalan SS Silverwood RJ Salman O Howard N. Associations between acute conflict and maternal care usage in Egypt: An uncontrolled before-and-after study using demographic and health survey data. Int J Heal Policy Manag. (2019) 8:158–67. 10.15171/ijhpm.2018.107 30980632 PMC6462197

[31] Rocanello-Snow K. The impacts of terrorism on maternal health in Afghanistan. Glob Sustain Develop Projects. (2021) 1. Available online at: https://scholarship.rollins.edu/sdg/1

[32] Alhassan GN Adedoyin FF Bekun FV Agabo TJ. Does life expectancy, death rate and public health expenditure matter in sustaining economic growth under COVID-19: empirical evidence from Nigeria? J Public Aff. (2021) 21:e2302. 10.1002/pa.2302 32904937 PMC7460965

[33] Chukwuma A Wong KLM Ekhator-Mobayode UE. Disrupted service delivery? the impact of conflict on antenatal care quality in Kenya. Front Glob Women's Heal. (2021) 2:9. 10.3389/fgwh.2021.599731 34816176 PMC8594042

[34] Wang G zhen. The Impact of Social and Economic Indicators on Maternal and Child. Health Soc Indic Res. (2014) 116:935–57. 10.1007/s11205-013-0330-y

[35] Zolala F Heidari F Afshar N Haghdoost AA. Exploring maternal mortality in relation to socioeconomic factors in Iran. Singapore Med J. (2012) 53:684–9. 23112022

[36] Bayati M Vahedi S Esmaeilzadeh F Kavosi Z Jamali Z Rajabi A. Determinants of maternal mortality in Eastern Mediterranean region: a panel data analysis. Med J Islam Repub Iran. (2016) 30:360. 27453890 PMC4934458

[37] Nikzadian A Agheli L Arani AA Sadeghi H. The effects of resource rent, human capital and government effectiveness on government health expenditure in organization of the petroleum exporting countries. Int J Energy Econ Policy. (2019) 9:381–9. 10.32479/ijeep.7575

[38] Anselin L Gallo JL Jayet H. Spatial panel econometrics. In: Mátyás L, Sevestre P, editors. The Econometrics of Panel Data Advanced Studies in Theoretical and Applied Econometrics, Vol 46. Berlin; Heidelberg: Springer (2008). 10.1007/978-3-540-75892-1_19

[39] LeSage J Pace RK. Introduction to Spatial Econometrics. Chapman and Hall/CRC. (2009).

[40] Elhorst JP Fischer MM Getis A. Handbook of Applied Spatial Analysis. Handb Appl Spat Anal. (2010) 377–407. 10.1007/978-3-642-03647-7_19

[41] Burridge P. On the cliff-ord test for spatial correlation. J R Stat Soc Ser B. (1980) 42:107–8. 10.1111/j.2517-6161.1980.tb01108.x

[42] Nwankwo CE. the effects of public health spending on maternal mortality in Nigeria. J Econ Sustain Develop. (2019) 9:141–52. Available online at: https://core.ac.uk/download/pdf/234648648.pdf

[43] Maruthappu M Ng KYB Williams C Atun R Agrawal P Zeltner T. The association between government healthcare spending and maternal mortality in the European Union, 1981-2010: a retrospective study. BJOG An Int J Obstet Gynaecol. (2015) 122:1216–24. 10.1111/1471-0528.13205 25492692

[44] Barlow P. Does trade liberalization reduce child mortality in low- and middle-income countries? A synthetic control analysis of 36 policy experiments, 1963-2005. Soc Sci Med. (2018) 205:107–115. 10.1016/j.socscimed.2018.04.001 29684913 PMC5956309

[45] Olper A Curzi D Swinnen J. Trade liberalization and child mortality: a synthetic control method. World Dev. (2018) 110:394–410. 10.1016/j.worlddev.2018.05.03429684913

[46] Bornemisza O Ranson MK Poletti TM Sondorp E. Promoting health equity in conflict-affected fragile states. Soc Sci Med. (2010) 70:80–8. 10.1016/j.socscimed.2009.09.032 19853342

[47] Ruiz-Cantero MT Guijarro-Garvi M Bean DR Martínez-Riera JR Fernández-Sáez J. Governance commitment to reduce maternal mortality. Health Place. (2019) 57:313–20. 10.1016/j.healthplace.2019.05.012 31146194 PMC6873917

[48] Wagner Z Heft-Neal S Bhutta ZA Black RE Burke M Bendavid E. Armed conflict and child mortality in Africa: a geospatial analysis. Lancet. (2018) 392:857–65. 10.1016/S0140-6736(18)31437-5 30173907 PMC6338336

